# The Preparation of High Saturation Magnetization and Low Coercivity Feco Soft Magnetic Thin Films via Controlling the Thickness and Deposition Temperature

**DOI:** 10.3390/ma15207191

**Published:** 2022-10-15

**Authors:** Wenjie Yang, Junjie Liu, Xiangfeng Yu, Gang Wang, Zhigang Zheng, Jianping Guo, Deyang Chen, Zhaoguo Qiu, Dechang Zeng

**Affiliations:** 1 School of Materials Science and Engineering, South China University of Technology, Guangzhou 510640, China; 2 Zhongshan R&D Center for Materials Surface and Thin Films Technology of the South China University of Technology, Gent Materials Surface Technology (Guangdong) Co., Ltd., Zhongshan 528437, China; 3 Yangjiang Branch, Guangdong Laboratory Materials Science and Technology Yangjiang Advanced Alloys Laboratory, Yangjiang 529500, China; 4 Institute for Advanced Materials, South China Academy of Advanced Optoelectronics, South China Normal University, Guangzhou 510006, China

**Keywords:** FeCo thin film, saturation magnetization, coercivity, thickness, deposition temperature

## Abstract

FeCo thin films with high saturation magnetization (4 π*M*_s_) can be applied in high-frequency electronic devices such as thin film inductors and microwave noise suppressors. However, due to its large magnetocrystalline anisotropy constant and magnetostrictive coefficient of FeCo, the coercivity (*H*_c_) of FeCo films is generally high, which is detrimental to the soft magnetic properties. Meanwhile, the thickness and deposition temperature have significant effects on the coercivity and saturation magnetization of FeCo films. In this paper, FeCo thin films with different thicknesses were prepared by magnetron sputtering at different temperatures. The effects of thickness and deposition temperature on the microstructure and magnetic properties of FeCo thin films were systematically studied. When the film thickness increases from 50 nm to 800 nm, the coercivity would decrease from 309 Oe to 160 Oe. However, the saturation magnetization decreases from 22.1 kG to 15.3 kG. After that, we try to further increase the deposition temperature from room temperature (RT) to 475 °C. It is intriguing to find that the coercivity greatly decreased from 160 Oe to 3 Oe (decreased by 98%), and the saturation magnetization increased from 15.3 kG to 23.5 kG (increased by 53%) for the film with thickness of 800 nm. For the film with thickness of 50 nm, the coercivity also greatly decreased from 309 Oe to 10 Oe (decreased by 96%), but the saturation magnetization did not change significantly. It is contributed to the increase of deposition temperature, which will lead to the increase of grain size and the decrease of the number of grain boundaries. And the coercivity decreases as the number of grain boundaries decreases. Meanwhile, for the thicker films, when increasing the deposition temperature the thermal stress increases, which changes the appearance of (200) texture, and the saturation magnetization increases. Whereas, it has a negligible effect on the orientation of thin films with small thickness (50 nm). This indicates that high-temperature deposition is beneficial to the soft magnetic properties of FeCo thin films, particularly for the films with larger thickness. This FeCo thin film with high saturation magnetization and low coercivity could be an ideal candidate for high-frequency electronic devices.

## 1. Introduction

With the development of the electronic information industry, the high frequency, miniaturization and integration are required for the electronic devices [1,2,3]. According to the Snoek’s limit, the initial permeability is inversely proportional to the ferromagnetic resonance frequency under a certain saturation magnetization [4]. Therefore, to obtain superior soft magnetic properties at high frequency, magnetic materials should have high saturation magnetization. Among commercial magnetic materials, the saturation magnetization of FeCo is the highest, reaching 24.5 kG when the Fe content is 65% [5]. The study by Najafi et al. also showed that the ratio of Fe and Co had a significant effect on the saturation magnetization, and the saturation magnetization reached the maximum when the Fe content was about 70% [6]. Moreover, according to Acher’s limit [7], the film material can exceed the restriction of Snoek’s limit, so FeCo films are ideal candidates for high-frequency electronic devices such as thin film inductors [8,9] and microwave noise suppressors [10,11]. However, due to the large magnetocrystalline anisotropy constant (~10 kJ/m^3^) and magnetostrictive coefficient (4~6 × 10^−5^), the coercivity of FeCo films is generally high, which is detrimental to the soft magnetic properties and hysteresis loss [12].

In order to reduce the coercivity of FeCo thin films, many studies have been investigated, such as adding under layer [13,14,15,16] or adding the third element [17,18,19] to change the microstructure of the film and reduce the coercivity. However, this method may lead to a decrease in saturation magnetization. Himalay Basumatary et al., studied the effect of deposition temperature on the magnetic properties of FeGa thin films and found that high temperature deposition can greatly reduce the coercivity, since high temperature deposition could be a feasible method to improve the soft magnetic properties of thin films [20]. There exist some studies on the effects of high temperature deposition on the magnetic properties of FeCo-based alloy thin films [21,22,23,24]. However, the relationships between structure and magnetic properties have not yet been systematically studied. Moreover, the above studies have only focused on thin films with relatively small thickness. However, in practical applications a certain thickness of the film is required to provide sufficient magnetic signal, and the thickness of the film has a great influence on the magnetic properties [25].

Therefore, in our work, FeCo thin films with different thicknesses were prepared by magnetron sputtering at different temperatures. The effects of thickness and deposition temperature on the microstructure and magnetic properties of FeCo thin films were systematically studied.

## 2. Experimental

**Figure 1:**
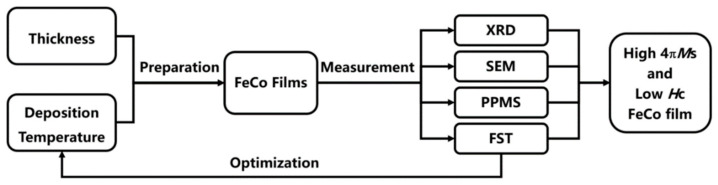
Flow chart for the preparation and testing of FeCo films.

FeCo target is purchased from AT&M Co., Ltd. (Beijing, China), and the composition is Fe: Co = 65:35 (atomic ratio). The Si substrates are purchased from Zhejiang Lijing Silicon Material Co., Ltd. (Quzhou, China) The experimental flow is shown in Figure 1. FeCo thin films with different thicknesses were deposited on Si (100) substrates using the DC magnetron sputtering technique under varying deposition temperatures. The base pressure of the magnetron sputtering system was 5 × 10^−4^ Pa. Sputtering pressure and power were kept constant at 0.8 Pa and 120 W, respectively, for all the depositions. Thin films with thicknesses of 50 nm, 100 nm, 200 nm, 400 nm, 800 nm and 1200 nm were deposited at room temperature. Additionally, thin films with thicknesses of 50 nm and 800 nm were deposited at different temperatures such as 25 °C, 200 °C, 300 °C, 400 °C, 425 °C, 450 °C, 475 °C, 500 °C, 525 °C and 550 °C. The crystal structure was analyzed by X-ray diffraction (XRD, Philips X’pert pro M, Amsterdam, The Netherlands). Hysteresis loops were detected with the Physical Property Measurement System (PPMS, Quantum Design PPMS-9), and the magnetic field is parallel to the plane of the film in the test. Surface morphology and cross-sectional morphology were characterized by scanning electron microscopy (SEM, SU8220, Hitachi Limited, Tokyo, Japan). Thin film stress is tested by film stress tester (FST, SuPro FST5000, Shenzhen, China).

## 3. Results and Discussion

**Figure 2:**
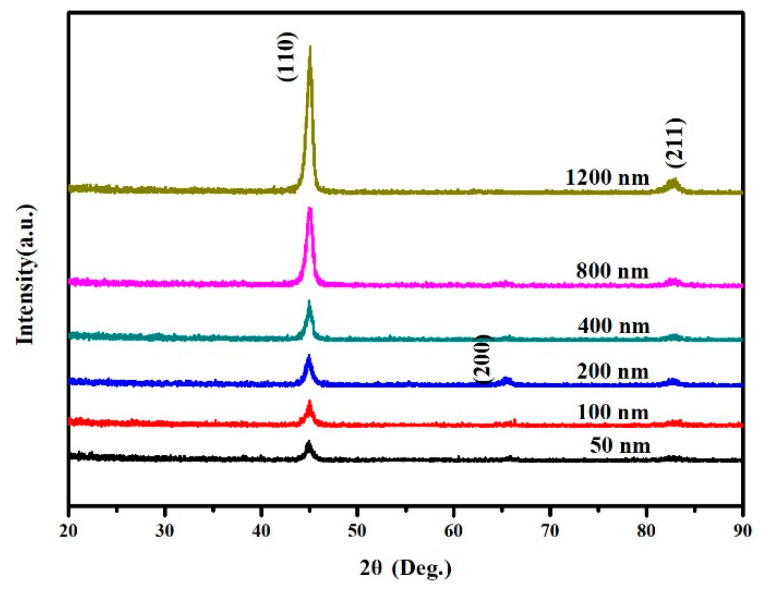
GI-XRD patterns for FeCo thin films with different thickness.

**Figure 3:**
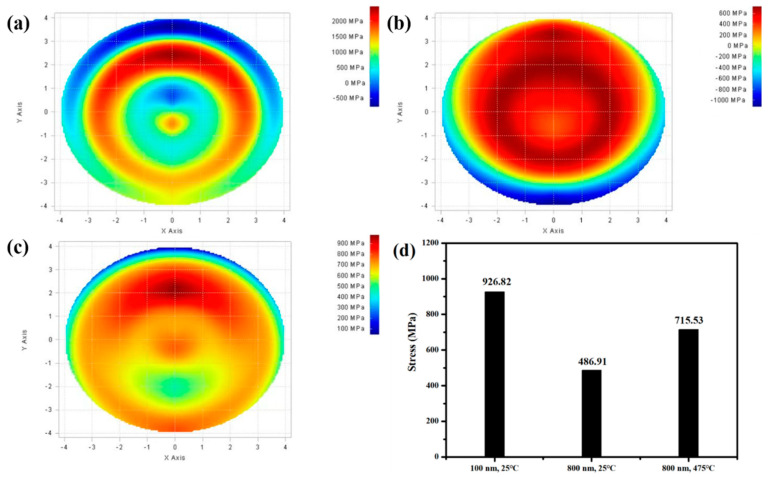
Stress distribution of FeCo thin films with different thicknesses and deposition temperatures: (**a**) 100 nm, 25 °C, (**b**) 800 nm, 25 °C, (**c**) 800 nm, 475 °C; and (**d**) the average stress.

Representative GIXRD patterns of FeCo thin films with different thicknesses are shown in Figure 2. It can be observed that the (200) diffraction peak gradually disappeared with the increase in thickness. This is due to the film stress decreasing with the increase in film thickness, as shown in Figure 3. Energy minimization is required during grain growth of thin films. When the film thickness is relatively larger, the stress of the film is relatively smaller and the surface energy is dominant. FeCo is body-centered cubic (bcc) structure, and the close-packed plane is (110). Therefore, to minimize the surface energy, the preferred orientation of the film is (110). When the film thickness decreases, the film stress increases. In addition to surface energy, strain energy also needs to be considered. Since the (200) texture of FeCo thin film is insensitive to stress, the surface energy increased by the transformation from (110) texture to (200) texture is less than the decrease in strain energy, resulting in the appearance of (200) diffraction peak [26]. The film stress decreases with increasing the film thickness, because during the film growth process the shrinkage of grain boundaries on the growth surface will lead to a tensile stress. However, with the increase in film thickness the grain size increases, resulting in a decreasing number of grain boundary, so the shrinkage decreases and the stress decreases [25].

**Figure 4:**
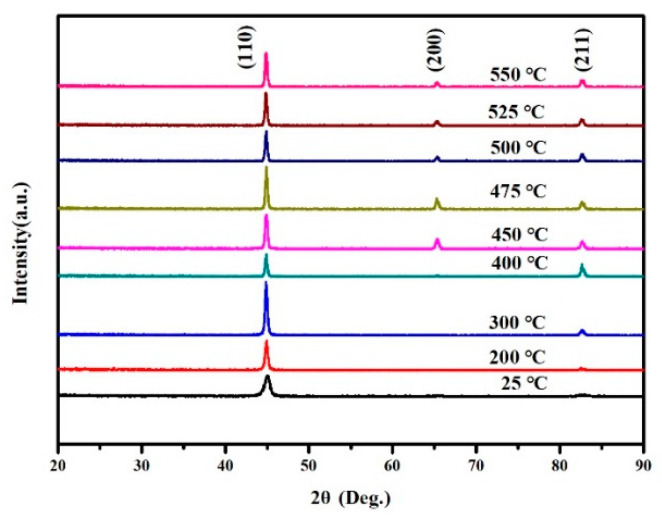
GI-XRD patterns for FeCo thin films with thickness of 800 nm deposited at different temperatures.

**Figure 5:**
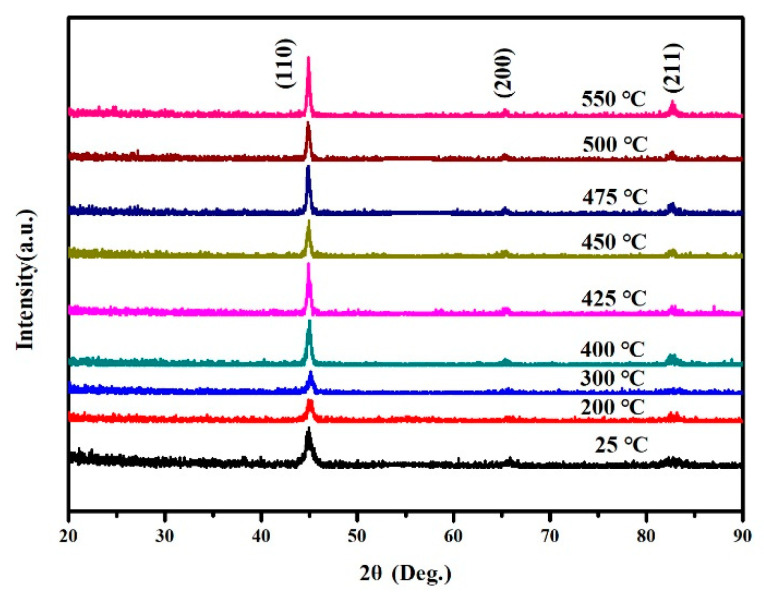
GI-XRD patterns for FeCo thin films with thickness of 50 nm deposited at different temperatures.

Figure 4 shows that for FeCo films with a thickness of 800 nm, when the deposition temperature increases, the (200) diffraction peak re-emerged. This is due to the thermal expansion coefficients of the film and substrate being different. During thin film deposition, the film and substrate are in a compatible deformation state at a specific temperature. When the film is deposited, both the film and the substrate return to room temperature. The change in temperature leads to an inconsistent shrinkage deformation of the film and the substrate, and the thermal stress will be generated in the film due to the discrepancy of the thermal expansion coefficient. As shown in Figure 3, when the deposition temperature increases from 25 °C to 475 °C the film stress increases from 486.91 MPa to 715.53 MPa. Therefore, to reduce the strain energy (200) texture appears in the film. For FeCo thin films with a thickness of 50 nm, it is different from the film with a thickness of 800 nm, the diffraction peak does not change significantly with the increase in temperature, and the (200) diffraction peak can be obtained when deposited at 25 °C, as shown in Figure 5.

**Figure 6:**
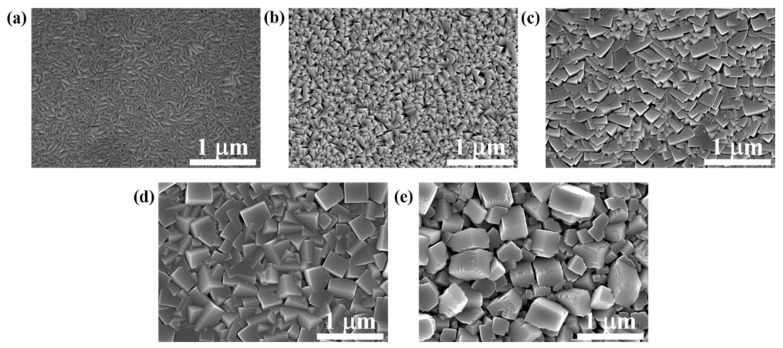
Surface morphologies of FeCo thin films with thickness of 800 nm deposited at (**a**) 25 °C, (**b**) 200 °C, (**c**) 400 °C, (**d**) 475 °C and (**e**) 550 °C.

**Figure 7:**
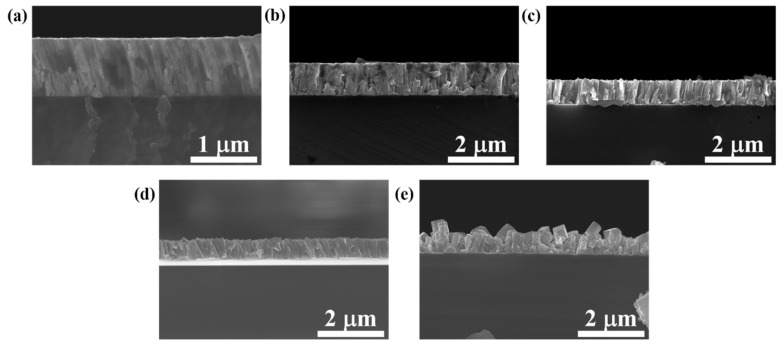
Cross-sectional morphologies of FeCo thin films with thickness of 800 nm deposited at (**a**) 25 °C, (**b**) 200 °C, (**c**) 400 °C, (**d**) 475 °C and (**e**) 550 °C.

**Figure 8:**
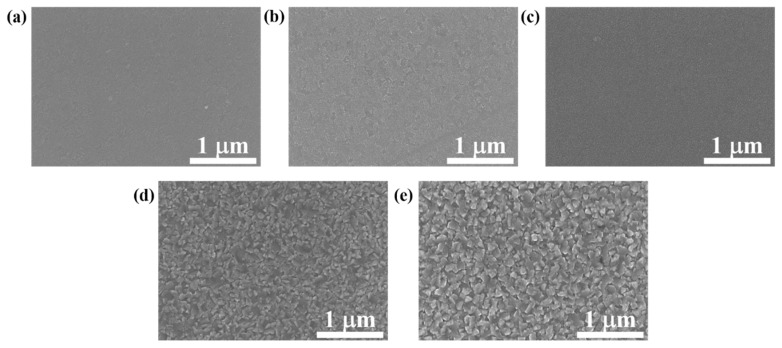
Surface morphologies of FeCo thin films with thickness of 50 nm deposited at (**a**) 25 °C, (**b**) 200 °C, (**c**) 400 °C, (**d**) 475 °C and (**e**) 550 °C.

In order to evaluate the effects of deposition temperature on the grain size variation, Figure 6 displays the surface morphologies of FeCo thin films with thickness of 800 nm deposited at different temperatures. It shows that as the deposition temperature increases, the grain size increases significantly. Relative works have already demonstrated that an increase in deposition temperature will lead to the increase in grain size of FeCo thin films [22,23,27]. The increase in deposition temperature will lead to the decrease in nucleation rate and the increase in grain growth rate. Under the comprehensive effect of the two factors, the grain size increases with the increase in deposition temperature [20]. Figure 7 shows that when the deposition temperature is lower than 475 °C, the surface roughness increases with enhancing the deposition temperature due to the increase in the grain size, but it is not obvious. However, when the deposition temperature increases from 475 °C to 550 °C, the roughness increases significantly, and the film density obviously decreases. For FeCo films with a thickness of 50 nm, the variation in microstructure with deposition temperatures is similar to that of 800 nm, except for the relatively small grain size, as shown in Figure 8.

**Figure 9:**
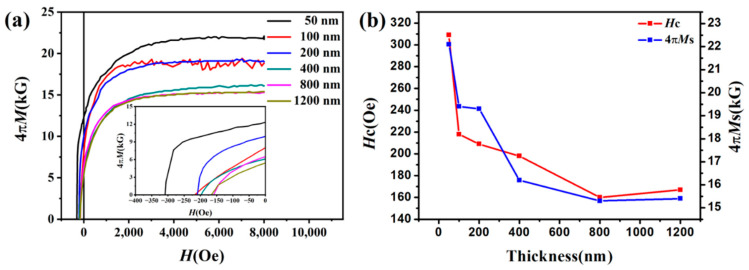
(**a**) Demagnetization curves of FeCo films with different thickness, and (**b**) dependence of the coercivity and saturation magnetization on thicknesses.

Figure 9 shows the variation in coercivity and saturation magnetization of FeCo films with different thicknesses. It indicates that the saturation magnetization and coercivity decrease first, and then remain stable with the increase in thickness. When the film thickness increases from 50 nm to 800 nm, the coercivity decreases from 309 Oe to 160 Oe, and the saturation magnetization decreases from 22.1 kG to 15.3 kG. The decrease in saturation magnetization is related to the disappearance of the (200) diffraction peak. Due to the effect of demagnetization field, the effective magnetization of the film should be located in the film plane. However, in practice there is an angle between the direction of effective magnetization and the film plane for FeCo film. Compared with (110) texture, the effective magnetization of (200) texture is closer to the film plane [28]. Therefore, the films with (200) texture would have higher saturation magnetization. Previous studies revealed that reducing film stress could significantly reduce coercivity [29]. This is because the stress in the film may lead to the magnetostrictive effect, resulting in magnetic anisotropy. As the thickness increases, the stress of the film decreases, so the coercivity decreases.

**Figure 10:**
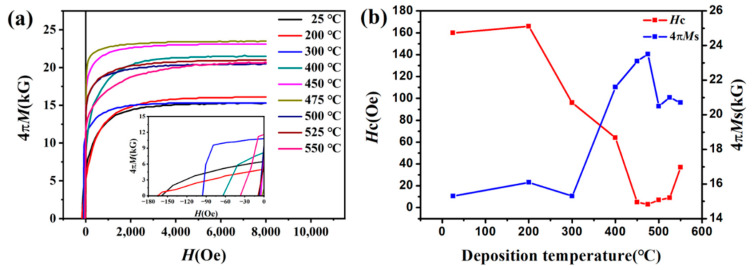
(**a**) Demagnetization curves of FeCo films with thickness of 800 nm deposited at different temperatures, and (**b**) dependence of the coercivity and saturation magnetization on deposition temperature.

In addition, the variation in coercivity and saturation magnetization of FeCo films with thickness of 800 nm and deposited at different temperatures are analyzed in Figure 10. When the deposition temperature is lower than 475 °C, the saturation magnetization increases and the coercivity decreases with gradually increasing the deposition temperature. The reason for the change in saturation magnetization is the same as mentioned above. When the deposition temperature increases, the thermal stress of the film increases, resulting in the existence of (200) texture in the film, and the saturation magnetization of the film increases. The decrease in coercivity is related to the change in grain size. The reverse magnetization process of soft magnetic materials is mainly realized by domain wall displacement. When increasing the deposition temperature, the grain size increases, the number of grain boundaries decreases, the blocking effect of grain boundaries on domain wall displacement is weakened, and the coercivity decreases. The greater roughness in the film gives rise to the surface defects, which can act as pinning centers to hinder domain movement, resulting in a higher coercivity [20]. When the deposition temperature is higher than 475 °C, the roughness of the film becomes even higher. At this condition, the effect of roughness on the coercivity of the film is dominant, so the coercivity begins to increase. Meanwhile, the saturation magnetization starts to decrease because the density of the film decreases.

**Figure 11:**
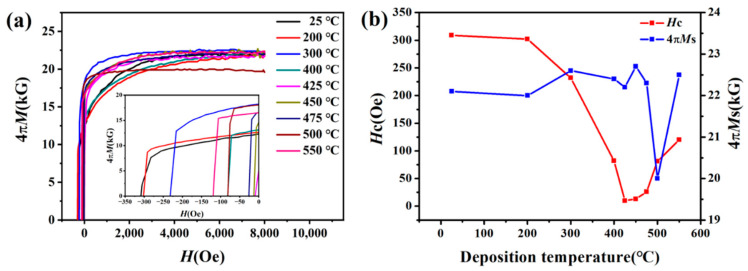
(**a**) Demagnetization curves of FeCo films with thickness of 50 nm deposited at different temperatures, and (**b**) dependence of the coercivity and saturation magnetization on deposition temperature.

Figure 11 shows the variation in coercivity and saturation magnetization of FeCo films with thickness of 50 nm deposited at different temperatures. When enhancing the deposition temperature, the variation trend of coercivity of FeCo film with thickness of 50 nm is similar to that of FeCo film with thickness of 800 nm, which decreases first and then increases. The coercivity is also closely related to the grain size and roughness. However, the saturation magnetization changes differently. The deposition temperature has little effect on the saturation magnetization of FeCo films with a thickness of 50 nm. It may be due to the FeCo films with thickness of 50 nm deposited at 25 °C also having (200) texture, and the deposition temperature has negligible effect on the orientation of thin films.

Comparing the results of Figure 10 and Figure 11, it can be seen that the effect of high temperature deposition on the magnetic properties of FeCo films with different thicknesses is not exactly consistent. For the FeCo film with a thickness of 800 nm, when the deposition temperature increases from 25 °C to 475 °C the coercivity decreases from 160 Oe to 3 Oe (decreased by 98%), and the saturation magnetization increases from 15.3 kG to 23.5 kG (increased by 53%). For FeCo thin films with a thickness of 50 nm, when the deposition temperature increases from 25 °C to 425 °C the coercivity decreases from 309 Oe to 10 Oe (decreased by 96%), but the saturation magnetization does not change significantly. This indicates that high-temperature deposition is beneficial to the soft magnetic properties of FeCo thin films, particularly for the films with larger thickness, and is available for future application in high-frequency electronic devices.

## 4. Conclusions

In summary, the effects of thickness and deposition temperature on the microstructure and magnetic properties of FeCo thin films were investigated. With the increase in film thickness, the decrease in stress leads to the disappearance of (200) texture, which reduces the saturation magnetization. When the film thickness increases from 50 nm to 800 nm, the saturation magnetization decreases from 22.1 kG to 15.3 kG. However, when the thicker film is deposited at high temperature, the increase in thermal stress leads to the reappearance of (200) texture, and the saturation magnetization of the 800 nm film increases from 15.3 kG to 23.5 kG. Additionally, (200) texture also exists in the thin films with small thickness deposited at room temperature, so the deposition temperature has little effect on the saturation magnetization. When the deposition temperature increases, the grain size obviously increases, resulting in a significant reduction in coercivity. For FeCo films with a thickness of 800 nm, when the deposition temperature increases from 25 °C to 475 °C the coercivity can be reduced by 98% to 3 Oe. This FeCo thin film with high saturation magnetization and low coercivity could be an ideal candidate for high-frequency electronic devices.

## Data Availability

Not applicable.
